# The social life of creative methods: Filmmaking, fabulation and recovery

**DOI:** 10.1111/1468-4446.13177

**Published:** 2024-12-27

**Authors:** Nicole Vitellone, Lena Theodoropoulou, Melanie Manchot

**Affiliations:** ^1^ AF Warr Senior Lecturer in Sociology Department of Sociology, Social Policy and Criminology University of Liverpool Liverpool UK; ^2^ Lecturer in the Sociology of Public Health Department of Public Health Policy and Systems University of Liverpool Liverpool UK; ^3^ Artist in Residence Centre for Health, Arts, Society and the Environment (CHASE) University of Liverpool Liverpool UK

**Keywords:** artistic methods, fabulation, filmmaking, recovery from drug and alcohol use, stigma and destigmatisation

## Abstract

In this article we consider the theoretical and methodological implications of Deleuzian fabulation for research on recovery from drugs and alcohol as an alternative way of making and doing methods in sociology. The article draws on data produced as part of an ongoing interdisciplinary research collaboration, begun in 2019, with the visual artist and filmmaker Melanie Manchot, social scientists Nicole Vitellone and Lena Theodoropoulou, and people in recovery from drugs and alcohol engaged in the production of Manchot’s first feature film STEPHEN. This project attends to the methodological practice of filmmaking as a way of thinking with and alongside colleagues from divergent disciplines about the role of methods, concepts and practices for confronting and resisting processes of stigmatisation. Investigating the research participants’ engagement with Manchot’s filmmaking practices in STEPHEN as a way to tell stories otherwise, our goal is to engage the social life of creative methods and in doing so, propose an alternative narrative of recovery. In this investigation, we use the term fabulation as developed by Deleuze. In *Cinema II*, Deleuze makes a distinction between the cinema of reality, where storytelling derives from the camera’s objective gaze and a given character’s subjective actions, and cinema verité where the boundaries between fiction and reality are blurred. In cinema verité, the camera is not an objective observer but an active producer that keeps reminding the viewer that the on‐screen characters are neither fully real, nor fictional. Attending to Deleuze’s description of fabulation as it emerges through this process of challenging the existence of ‘real’ identities in cinema, and beyond, we investigate the use of cinematic devices and fabulative processes of filmmaking in the production of STEPHEN. In doing so, the article develops a methodological account of the activity of fabulation as a material and embodied practice that resists processes of stigmatisation. Through this interdisciplinary project, we propose a new arts‐based research agenda which points to the ways in which fabulation as a minor mode of recovery concerns an engagement with the creation of a people to come.

## 1 INTRODUCTION

In this article we investigate collaborative filmmaking and the impact of fabulation in cinematic narrative as a practice that inspires social scientists to rethink the position of the arts in thinking sociologically. Drawing on a four‐year interdisciplinary research project starting in November 2019, involving the visual artist and filmmaker Melanie Manchot, social scientists Nicole Vitellone and Lena Theodoropoulou, and people in recovery from drugs and alcohol from Liverpool community arts groups engaged in the production of Manchot's first feature film STEPHEN (2023), we address the deployment and use of the camera in the process of filmmaking. We use the camera as a methodological device for thinking about how dealings with colleagues from divergent disciplines and the community can lead to new concepts and methods that interrupt and intervene in the practice of sociological research. Attending to the making of STEPHEN in the production (filming) and distribution stages (art installation at Liverpool Biennial, 2023), the article investigates the process of filmmaking as a mode of knowledge production that forces us to rethink the power of artistic methods and practices as creating new realities. In thinking with the methods mobilised in and through the making of STEPHEN (2023) as a site for sociological inquiry, we take seriously Savage (2013) and Law et al.’s (2011) agenda on the social life of methods as a lens to investigate the affordances mobilised through methods. For Savage (2013) and Law et al. (2011), methods are both fully of the social world and active in making it. The operations embedded in methods are recognised not just as technical tools deployed by academics to represent reality, but methodological devices constituted by advocates outside of the academy that question the divisions between theory and method, and open new ways of understanding the relationship between the social, the cultural and material. The challenge for the social sciences, is to develop tools to handle the life and liveliness of methods constituted by non‐academic advocates, think about what methods are doing, the data they're making, and their capacity to constitute and intervene in the social (Law et al., 2011).

Responding to this challenge to embed sociological investigation in non‐academic methodological practices, we focus on Manchot's process of filmmaking in STEPHEN (2023) as a case study for questioning the methods of artists as both social and active in intervening in the world. However, we recognise following Mair et al. (2016), that enquiring into the social life of creative methods can only be a starting point for investigating the practice of filmmaking as social. The danger of treating methods as social and cultural phenomena independently of the activity of the researcher and the practical doing of research, they warn, is that it encourages ‘methodological hagiography’ where the lives of ‘great men’ are replaced with the lives of ‘great methods’ (Greiffenhagen et al., 2015). What's required to investigate the double life of methods in terms of their value for research and understandings of the social, they argue, is in depth studies of hands‐on research practice demonstrating the utility of methods in use (Greiffenhagen et al., 2015). In taking up the call to address the doing of methods in situ the article responds to the interdisciplinary turn in Science and Technology Studies (STS) to take seriously artistic practices, concepts, collaborations and experiments as material forms of knowledge production that require empirical engagement with their making to better understand how the arts contribute to the investigation of more than human worlds (Borgdorff et al., 2020; Dennis, 2019; Ledesert, 2022; Rogers, 2022; Rogers et al., 2021, 2023; Sormani et al., 2019).

In the first part, we historicise the sociology of recovery, highlighting the uses of Goffman's (1959, 1963) concepts of stigma and dramaturgy, and their links to methods of data production and analysis in academic research. Treating Goffman's methods as objects of analysis that force us to rethink the relationship between theory and method, we address the role and influence of the arts in opening interdisciplinary approaches of inquiry that take seriously the affordances of filmmaking as a specific topic of sociological research. In the second part, we turn to Manchot's description of the fabulative devices and methods of filmmaking in the production of her film STEPHEN (2023) and investigate their constitutive and transformative potential. Describing her use of the camera, Manchot attends to the capacity of creative methods to intervene in the social in ways that disrupt the divisions between fact and fiction, real and imaginary, collective and individual, and artistic and social scientific knowledge practices as separate matters of concern. In the third part, we consider the affordances mobilised in and through Manchot's artistic methods of filmmaking in the production and installation of STEPHEN. Drawing from observational data and interviews with participants involved in the making of STEPHEN, we make explicit the link between fabulative filmmaking as described by Deleuze (1989) and creative methods of knowledge production, attending to the effects and consequences of fabulation for processes of destigmatisation and recovery. In extending the critical agenda associated with the social life of methods to artistic practices, visual production and empirical sources we show how an interdisciplinary research agenda that attends to the process of filmmaking involves the making of new realities and creation of a people to come.

### 1.1 What's wrong with the sociology of stigma?

The contribution of Goffman's (1963) theory of stigma to the social sciences has been widely acknowledged for producing a range of interventions including: researching the meaning of stigma in affected communities, investigating the impact of stigma in science, social policy, legal, medical and media discourse; and attention to the different tools and devices academics, artists, experts and activists engage in as strategies of destigmatisation in practice. While Goffman's (1963) theory of stigma has been deployed in a broad range of studies, including the field of harm reduction and critical drug studies to address the persistence of addiction stigma in relation to people who use alcohol and drugs (see for example, Farrugia et al., 2021; Fraser et al., 2017; Fraser et al., 2020; Sutherland et al., 2024; Treloar, Lancaster, et al., 2022; Treloar, Cama, et al., 2022), the use of the stigma concept and use of sociological methods in stigma research has also been rethought and contested. Calling into question the impact of Goffman's (1963) intervention as a way of seeing, classifying and understanding discriminatory attitudes and practices, and the contention that stigma can be ameliorated, Tyler and Slater (2018) argue that what is often neglected in sociological research are ‘structural questions about the social and political function of stigma as a form of power’ (p. 729). What's required to rethink stigma more sociologically, they argue, is to engage with ‘where stigma is produced, by whom and for what purposes’ (p. 721). The trouble with the sociology of stigma, according to Tyler and Slater (2018), is a limited understanding of the ‘importance of the role of symbolic structures and social mediating agencies in the production of inequality and marginality’ (p. 735). By drawing on the expertise and theoretical resources of stigma theorising explored within the visual arts, Tyler and Slater (2018) seek to reverse the marginalisation of non‐academic research in sociology and highlight how stigma is resisted through a range of methodological approaches.

In rethinking the sociology of stigma in relation to questions of power and structure, Tyler and Slater's (2018) critical methodological intervention builds on Parker and Aggleton's (2003) earlier interrogation of the conceptual adequacy and usefulness of Goffman's framework of stigma in relation to HIV and AIDS, and the limitation of individualised analyses of stigma for producing effective interventions as a resource of social change. To better respond to stigmatisation and discrimination as social processes, Parker and Aggleton (2003) call for a new agenda for research that involves three lines of inquiry, including new conceptual studies, new investigative studies, and strategic and policy‐oriented research, to produce interventions that contribute to programmes and policies that reduce suffering. Developing conceptual lines of enquiry requires interrogating the adequacy of existing concepts and the identification and development of new ideas and categories of thinking that allow for a new vision and new role for theory in understanding the nature of social processes of stigmatisation. In order to rethink epistemological frameworks and research designs, Parker and Aggleton (2003) call on social scientists to seek inspiration not from literature, but community participation and Goffman's (1963) insights concerning the impact of stigma in the construction of a spoilt identity.

Developing a research agenda which takes inspiration from Goffman's (1963) insights on stigma, McIntosh and McKeganey (2001) define recovery from dependence on illegal drugs as involving the repair of a stigmatised or spoilt identity. Drawing on Goffman (1963) and Biernacki's (1986) theory of the recovery process as involving a ‘mental dissociation’ in the conceptualisation of one's identity, McIntosh and McKeganey (2001) argue a key feature of successful recovery involves restoring a spoilt discredited identity by recreating old identities and/or inventing new ones. Yet, while Goffman's concept of stigma and qualitative methods are deployed in McIntosh and McKeganey's (2001) sociological research to address the process of recognising and acknowledging a spoilt identity in initiating recovery from drugs and alcohol, Neale et al. (2011) question the usefulness of Goffman's text *Stigma* (1963) as a starting point for engaging the social process of recovery. The trouble with repairing a damaged sense of self, Neale et al. (2011) argue, is it essentialises and individualises the recovery process and disempowers and stigmatises people who use drugs. Proposing an alternative theoretical and methodological framework that sidesteps the limitations of stigma theory for understanding recovery, Neale et al. (2011) shift their engagement with Goffman's work from the concept of spoilt identity in *Stigma* (1963) to dramaturgy in *The Presentation of Self in Everyday Life* (1959). Focusing on Goffman's theatrical sense of dramaturgy, as the act of a performer enacting different performances to different audiences, Neale et al. (2011) suggest the changeable nature of identity emphasised by Goffman's preoccupation with the theatre in *Presentation* (1959) offers ‘a quasi harm reduction approach to identity work’ (p. 5). Taking Goffman's theory of dramaturgy, metaphor of the theatre, and preoccupation with drama and performance as a useful methodological framework for understanding the process of recovery as achieved in social interaction with others, Neale et al. (2011) provide insights for a treatment approach that both avoids pathologising addiction as a spoilt label of trapped individuals and recognises the possibilities of doing recovery in positive ways that may not be totally abstinent. Although dramaturgy can be used to encourage the exploration of positive roles and preformative identity construction of the presentation of self beyond ‘spoilt’ and ‘unspoiled’, Neale et al. (2011) conclude, ‘we still seem unclear on how this happens and how recovery might be best promoted’ (p. 8). What's required, they argue, are other postmodern theoretical approaches developed since Goffman's writings that might develop these understandings further.

### 1.2 Goffman, interdisciplinary methods and make believe

Whilst Goffman's conceptual analyses in *Stigma* (1963) and *Presentation of Self in Everyday Life* (1959) have been reimagined to understand social processes of stigmatisation and recovery, we would also point to the ways the privileging of concepts of stigma and dramaturgy in sociological research have blocked social scientists from engaging with Goffman's methods and his interdisciplinary methodological practice. What distinguishes Goffman's research methods is that they are not limited to descriptions of the normal and the stigmatised in naturally occurring interactional situations. They also include imaginary and fictional figures and stories in literary texts, documentary realism, drama and cinema, as sources of empirical data (Smith, 2006, 2022). Drawing attention to Goffman's interdisciplinary methodological practice of cutting texts up into strips of activity, Love (2013) points out Goffman's use of literary and nonfiction texts and fabricated anecdotes as documentary sources allow him to make a ‘wide range of situations available for analysis and study’ (p. 427). The method of cutting reality into strips of behaviour allows Goffman to see stigma ‘as a property of a scene, not of the person in it’ and thus ‘deontologize stigma’ (Love, 2013, p. 426). While the procedures embedded in Goffman's method call into question the divisions between fiction and reality, humanities and social science research methods, and textual and empirical analysis, Love (2013, 2019) points out Goffman's postmodern interventions have been largely overlooked by humanities scholars as potentially useful tools for critical analysis. Responding to Love's (2013, 2019) observation of what Goffman's methods do, the data they make and their capacity to constitute the social, we want to stress that the interdisciplinary inspiration of Goffman's methodological approach in *Stigma* (1963) and *Presentation* (1959) has also been largely sidelined in the social sciences.

Mapping an interdisciplinary terrain for sociology that takes the theatrical stage, art and cinema as empirical sources of sociological inspiration has been central to the work not only of Goffman (1959, 1963), but of other sociologists including Mills (1959) and Becker (1982, 2007). Learning from the practices of novelists and artists is seen as central to developing an imaginative methodological framework that brings into view relations between the individual and the social that had gone unnoticed (Gane & Back, 2012). Smith (2022) points out, that by looking outside the academy and boundaries of the discipline Goffman was able to develop methods that inform his theoretical contribution not only in relation to *Stigma* (1963), but the conceptual articulation of the dramaturgical in *Presentation of Self* (1959), which includes elements of make believe (theatrical gestures, daydreaming, joking) extensively developed from fictional sources, especially drama and film in his later work *Frame Analysis* (1974). By developing a research method for analysing make‐believe as part of, rather than a separate province of meaning from everyday sense making activities, Smith (2022) notes Goffman's *Frame Analysis* (1974) aims to illustrate the intimate relationship of make‐believe to reality as ordinary and recommends ‘a close study of each in order to learn about the other’ (Smith, 2006, p. 60). What stands out in *Frame Analysis* (1974), according to Smith (2006), is Goffman continues the theme of dramaturgy, returning to the concept of performance and staged and unstaged activity. What's sociologically significant about Goffman's frame perspective, Smith (2006) argues, is the shift in focus away from the analysis of the interactional order in *Stigma* (1963), towards the individual's experience and how they interpret and make sense of strips of activity. While imaginary and fictional data play a key role in the situational perspective of frame analysis, Smith (2022) highlights how Goffman's method of using strips of data taken from novels, drama and film as part of *ordinary* behaviour is deemed not to have the same empirical authority gained from qualitative social research methods of investigating everyday interactions.

### 1.3 Deleuze, filmmaking and the power of the false

In what follows we seek to reclaim the value of artistic methods in Goffman's interdisciplinary methodological approach and his insistence that social research methods do not have epistemic authority over artistic practices of knowledge making. In so doing, we take seriously Goffman's interdisciplinary analysis of make believe as an ordinary activity in everyday life and address its sociological relevance. Focusing our investigation on the artistic practice of filmmaking, the fictions invented by filmmakers, and their social effects as sources of data, we attend to the relationship between fictional activities and reality. Our analysis is informed by Deleuze's (1989) concept of fabulation in his description of filmmaking. The concept of fabulation was originally developed by Bergson to describe the ‘paradox of fiction’, meaning ‘the problem why we feel real emotions for unreal (fictitious) people and the events that befall them’ (Mullarky, 2007, p. 54). Bergson's answer is that fiction, and especially filmed fiction, makes events come alive (ibid.). With fabulation, Bergson argues, we are not dealing with imagination, simulation or pretence, but with ‘the reactions of [people] to [their] perception of things, of events, of the universe in general’ (Bergson, 1977, p. 162, cited in Mullarky, 2007, p. 55). Bergson also acknowledged that fabulation could be an ‘interesting pathology’ echoing the views of psychologists and psychiatrists who described fabulation (and confabulation) as an unintentional production of false propositions (Stenner, 2018, p. 172).

Deleuzian fabulation draws on the Bergsonian one. In *Cinema II*, Deleuze (1989) deploys fabulation to complicate the filming event by addressing the difference between the cinema of reality, where storytelling derives from the camera's objective gaze and a given character's subjective experience, and cinema verité, where the camera is not an objective observer but an active producer that keeps reminding the viewer that the on‐screen characters are neither fully real, nor fictional. Fabulation, for Deleuze (1989), emerges through this process of challenging the existence of ‘real’ identities in cinema. In later writings, Deleuze (1995) takes fabulation further by stressing the importance of politicising the term by highlighting the relevance of minority narratives. Stenner (2018) points out that for Deleuze, the social life of cinematic devices is of ‘direct social relevance’ since it ‘“contributes to the invention of a people”’ (p. 175). Through cinematic practice ‘clear identities are scrambled as they melt down and are reconstituted’ (p. 176). Instead of affirming coherent identities, the activity of storytelling enabled by fabulation ‘summons a zone of indiscernibility between “characters” and the “film maker”’ (p. 175). The artist plays a central role in this process of the metamorphosis of the true as a transformed relation between fiction and reality: they are ‘the creator of truth, because truth is not to be achieved, formed or reproduced; it has to be created’ (Deleuze, 1989, p. 152).

Echoing the need to unpack the political meaning of fabulation, feminist scholars drawing on Hartman (2008) and Haraway (2021) writings, have deployed speculative fabulation to describe and enact world‐building practices (see for example, Carstens, 2020; Hiltunen & Campbell, 2024; Lizárraga, 2023) and in some cases brought together Deleuze and speculative fabulation. Coleman's (2020) interdisciplinary study of *Glitterworlds*, for example, highlights the uses of bringing into dialogue different approaches with the social sciences, especially STS, to account for the way fantasies fabricated through fabulation are not separate from real life but blurred together. In order to open a dialogue on the imaginary fictions crafted through the artistic practice of fabulative filmmaking, its connection to the constitution of real life, and relevance for social science and STS research (Borgdorff et al., 2020), in the next section we expand and make explicit the use of fabulative methods in the making of STEPHEN (2023). Following Michael's (2022) suggestions, on how to operationalise the potential future orientation of fabulation for sociological research; by attending to how fabulation ‘is “embodied” in, or enacted through, existing cultural artefacts and practices’; ‘disseminated or “transmitted” through scholarly and engaged works and practices’; and ‘as a part—a prompt and/or product ‐ of participant engagement’ (p. 83), we engage the sociomaterial practices of crafting and telling fables in the film STEPHEN. Turning to Manchot's description of her visual methodological practice as a filmmaker we offer a way of thinking about the social relevance of fabulation. Focusing on the active effect of the camera in producing fabulative narratives that are transformative of fixed identities, we seek to emphasise how new modes of being emerge with and through the camera as a relational device fully of the social world, and active in making and transforming it in ways that disrupt labels and challenge addiction stigma.

### 1.4 STEPHEN and fabulative filmmaking

I hate to be labelled.

An addict! What is that? I mean, I’m so much more than just an addict. Hm… But there you go, that’s what people want to see, so people see that.

**FIGURE 1:**
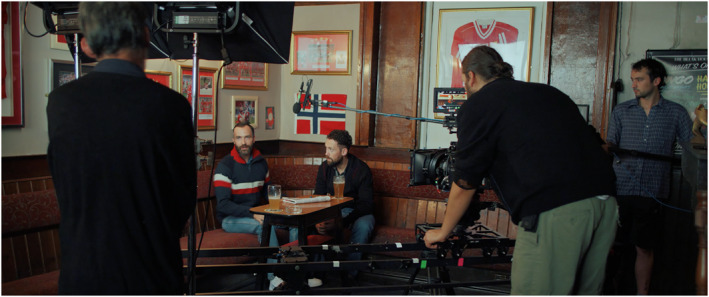
STEPHEN, film still, 2023. *Credit*: © Melanie Manchot and STEPHEN Film Ltd, commissioned by Liverpool Biennial.

This is what one of the members of the support cast^1^ says in STEPHEN (Timecode: 00.44.24): a hybrid documentary/fiction feature film made with people in recovery from drugs and alcohol. This brief monologue, spoken directly to camera, occurs in a roundtable discussion where a group of people come together in what could be a script read‐through, a recovery meeting or a group therapy session. What this scene is in content, form and genre is deliberately left open to interpretation, and it sits on the cusp between the real and the fictional, between speaking from lived experience and speaking in a mode of fabulation. It is real as we encounter people, not scripted characters, speaking their mind on the question of labels and stigma, on being called ‘an addict’. However, this is not cinema of reality per se, nor strictly documentary but fabulative filmmaking, as the cast are performing real‐life in a situation that is clearly and openly designated as a filmed environment, with the camera and crew visible at various points in the frame (see Figure 1). Here, the cast produce and perform versions of themselves towards a camera physically situated at the centre of the discussion and which people at times address directly. The situation and the material filmed is quasi‐documentary as the camera observes a situation that plays out in real time, in one continuous long take. Yet it is also a stage, a set‐up, whereby the active presence of the camera affects and potentially transforms the stories told. The scene becomes fabulative as it oscillates between fact and fiction to explore innovative forms of narrative storytelling that free the teller from the constraints of either truth or script.

**FIGURE 2:**
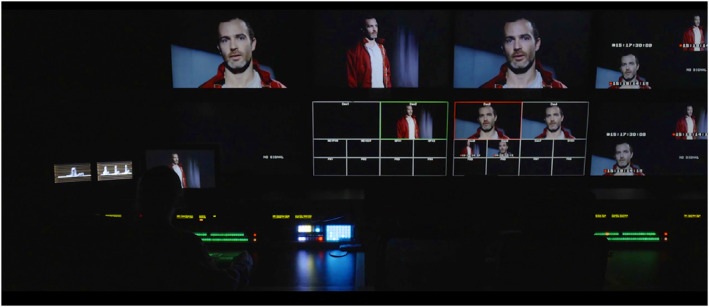
STEPHEN, production still, 2023. *Credit*: © Melanie Manchot and STEPHEN Film Ltd, commissioned by Liverpool Biennial.

In terms of its form, STEPHEN is both an art installation comprising a multi‐channel installation, and a feature film of 78 min, which is shown at film festivals, in cinemas, pop‐up screenings and gallery/museum presentations. Its multiplicity of presentation formats and outlets is part of its strategic positioning as an unfixed creative object. The film focuses on Ste (Stephen Giddings) who appears in practically every scene, both as himself and as his fictional character, Thomas Goudie (see Figure 2: Stephen auditioning to play Thomas). Stephen Giddings grew up with and around addictions. STEPHEN is a film‐within‐a‐film that combines narrative fiction with real‐life observation and archive material. It takes us on intimate journeys into two characters as Ste auditions for and takes on a role in a fiction film. As fiction merges with reality the separation between person, actor, and character dissolves.

Produced with a mixed cast of people in recovery alongside four professional actors, the film's main cinematic language is Steadicam with extended long takes pointing up the borders where performances begin and end. Within this visual practice cameras are never neutral technical devices, nor are they purely objective, as the characters actively engage with them during the filming process. Their acknowledged, physical presence charges and changes people's actions, gestures and speech acts, hence foregrounding that there is no neutral or passive position for the camera. This specifically applies once there is direct performance‐to‐camera, which entails a process of producing versions of self towards this recording ‘eye’.

## 2 FABULATION WITHIN THE STORIES OF STEPHEN

STEPHEN is based on multiple real stories, retold, reconstructed and re‐enacted. It starts with Stephen Giddings and his real‐life stories, retold in the film as interviews in which Stephen plays a version of himself directly to camera. These interview sections form the spine of the film, filmed with a fixed camera, and a static set up that resembles a dressing room. Significantly, when Stephen looks into the camera, he looks at me, the filmmaker, through a series of mirrors set up in such a way that he appears to face the camera. This device, called an interrotron, is a variation of the teleprompter and is often used to interview subjects and create a sense of direct engagement. Using the interrotron as a methodological device rather than simply a technological tool, sets up a fiction that prompts Stephen to enact a version of himself not just to camera but also towards his director and collaborator. Stephen tells his stories to Manchot, the director, not a disembodied camera lens, and Manchot's reaction ‐ via a set of mirrors ‐ encourage his performance.

**FIGURE 3:**
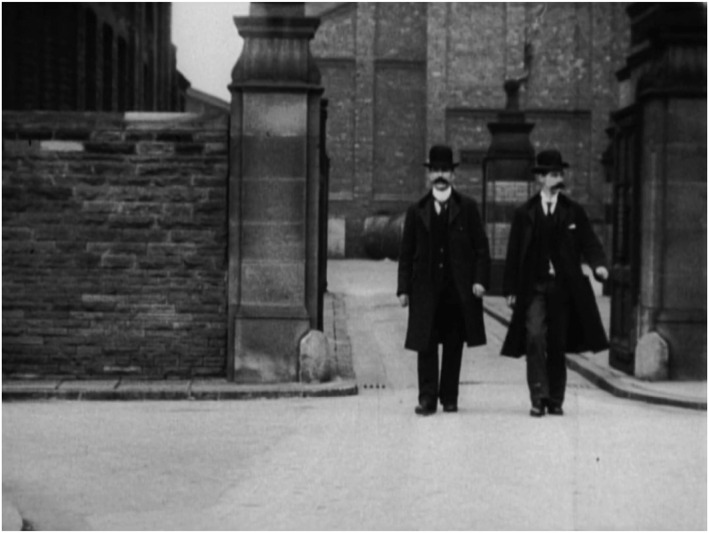
Arrest of Goudie, film still, 1901. *Credit*: Courtesy of British Film Institute (BFI) Archive, licensed to STEPHEN Film Ltd.

A film‐within‐a film, STEPHEN is based on Arrest of Goudie (1901), held in the British Film Institute archive (see Figure 3). *Arrest of Goudie* is the first ever crime‐reconstruction and first movie made in Liverpool, Stephen's hometown as well as that of the support cast. It tells the real‐life story of Thomas Goudie, a clerk at the Bank of Liverpool, caught embezzling £170,000—the equivalent of £22million in today's value—to cover his spiralling gambling debts. Victorian filmmakers Mitchell and Kenyon made their short film days after the real‐life arrest, with local people enacting the roles of Goudie, his landlady, police officers and bystanders. This re‐enactment was shown to local people within a day or two of the making, playing on the desire of people to either see themselves or their local geography flicker in front of their eyes in a darkened room. In STEPHEN, the Thomas Goudie story is re‐imagined as a near‐contemporary film‐within‐a‐film for which Giddings auditions, and in which he takes on the principal role of Goudie. A reconstruction of a reconstruction, STEPHEN asks Giddings and the entire support cast to use their lived experience of substance use to fabulate the story of a gambling addict.

**FIGURE 4:**
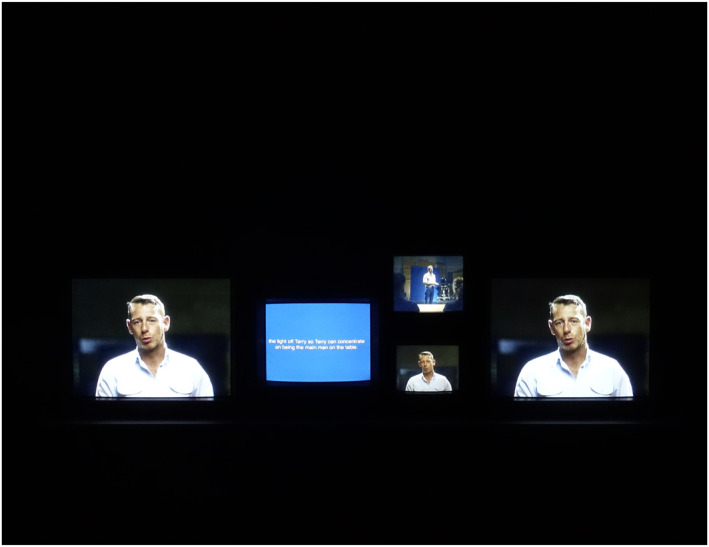
STEPHEN, installation view, Liverpool Biennial, 2023. *Credit*: © Melanie Manchot and STEPHEN Film Ltd, commissioned by Liverpool Biennial.

The contributions of our support cast involve them becoming multiple personas within STEPHEN, as each of them is there as themselves and as a fictional character. This is particularly pronounced in the multi‐channel installation that is the work's artworld, as opposed to the cinematic iteration. In the multi‐channel presentation, the support cast are seen auditioning for their roles, going through costume fittings, and appearing in front of the camera ‘in‐character’ to speak about their ‘actual’ self, telling stories about themselves from the position of their fictional character (see Figure 4). It is here, as it is in Stephen Giddings' interview sections, that fabulation emerges, as the cast is freed from the demands to either relate actual events or follow a script. They speak through the camera to the audience both in language and gestures created for their fictional characters, about stories that emerge from their lived past. In the process, they imagine and embody new ways to live and experience those memories, and creatively shape these past becomings towards the camera and towards themselves in the present and the future.

## 3 FABULATION IN THE GENRE‐DEFYING SHAPE OF STEPHEN

**FIGURE 5:**
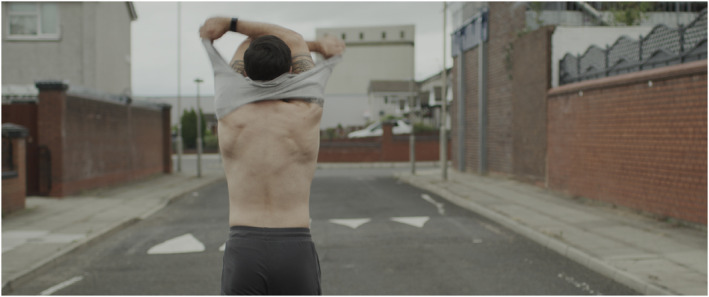
STEPHEN, film still, 2023. *Credit*: © Melanie Manchot and STEPHEN Film Ltd, commissioned by Liverpool Biennial.

**FIGURE 6:**
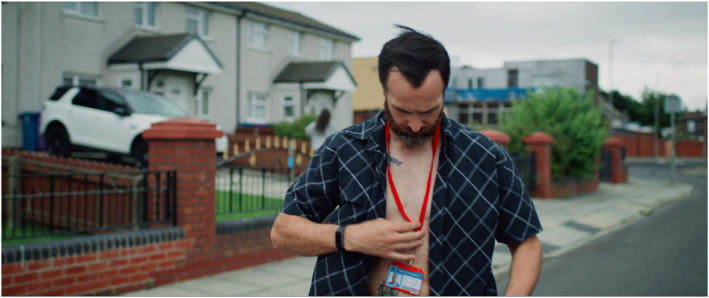
STEPHEN, film still, 2023. *Credit*: © Melanie Manchot and STEPHEN Film Ltd, commissioned by Liverpool Biennial.

STEPHEN was made as a genre‐defying film that productively embraces the slippages between cinematic languages that are often seen as distinct or even mutually exclusive. STEPHEN unfolds as a continuous slippage between sections of original archive material from 1901, direct‐to‐camera interviews, observation, and a scripted story filmed cinematically. Stephen is often seen walking, and in one scene he parades down a residential street in Liverpool, stripping off his clothes and taking on various items of costume, trying them on, keeping some and discarding others (see Figures 5 and 6). He walks out of his ‘actual’ self and into his ‘fictional’ character. Far from affirming coherent identities, this activity of fabulation, as Deleuze (1995) describes, summons a zone of indiscernibility between the character and the filmmaker.

Both character and filmmaker are involved in a process of ‘“making up” in which who they are becomes inseparable from the before and the after of a “passage from one state to another”’ (Stenner, 2018, p. 175). The metamorphosis enabled by fabulation is not inconsequential but of direct social relevance, since it ‘contributes to the invention of a people’ (Stenner, 2018, p. 175); and in the process the invention of a new way to produce drug use and recovery narratives. Importantly, the fabulative processes occurring around the camera take effect on both sides of the lens, yet also offer a respite from the categorical separation of fact and fiction.

In STEPHEN, situations where the categories of fact and fiction become productively entangled situate the cast and crew in a matrix of fabulative visual storytelling, opening the possibility to become other, without the need to dispose of the self and purely take on a role. This fabulative activity is particularly evident in one of the three elements in the installation, a two‐channel work called ‘The Costume Fittings’. During ‘The Costume Fittings’ each member of the cast enters a specially created ‘photobooth’ set‐up where they step in front of a one‐way‐mirror and are asked to observe themselves. A one‐way mirror is a reciprocal mirror that appears reflective on one side and transparent at the other. The camera films through the mirror as if it were glass, while the person in front looks at the mirror without seeing the camera. The cast do this twice: once in their day‐to‐day personal outfit and then again, hours later, after their costume fitting. Some talk, some observe in silence. Here, in subtle ways, we observe the process of ‘an invention of a people’ unfold before us. What we see is neither observation nor fiction but a space in between, where each member of our cast fabulates two characters. What is significant, is how the camera merges with the device of the mirror in which the participants see themselves, while simultaneously producing themselves. In this set‐up, the mirror is not a Foucault (1975) panoptic tool of self‐surveillance, but a fabulative methodological device that invites each cast member to use its reflective qualities to fabulate a new potential, for themselves towards the camera, and towards the film's audiences.

Whilst the use of cameras and the apparatus of cinema is deployed here to open opportunities for becomings that resist and challenge processes of stigmatisation, this is not to deny that fabulation and the power of cinematic narration can also be subverted towards detrimental means and purposes. One prime example is the highly problematic subgenre of Bergfilm, The Blue Light (1932), produced by Leni Riefenstahl during the Nazi regime, which highlights the power of cinema to promote fascism (Krakauer, 1995; Morris, 2012). While our interdisciplinary research project and context‐specific engagement with fabulation has led to an emphasis on its mobilisation as a positive force, according to Spinoza, ethics does not entail the attachment of ‘positive or negative values to actions based on a characterisation or classification of them according to a pre‐set system of judgement. It means assessing what kind of potential they tap into and express’ (Massumi, 2002, p. 217, cited in Theodoropoulou, 2023). Our engagement with the practice and activity of fabulation, as it emerged in the making of STEPHEN, expresses the potential of thinking with the cinematic devices as destigmatising forces.

## 4 FABULATION IN THE INSTALLATION OF STEPHEN

**FIGURE 7:**
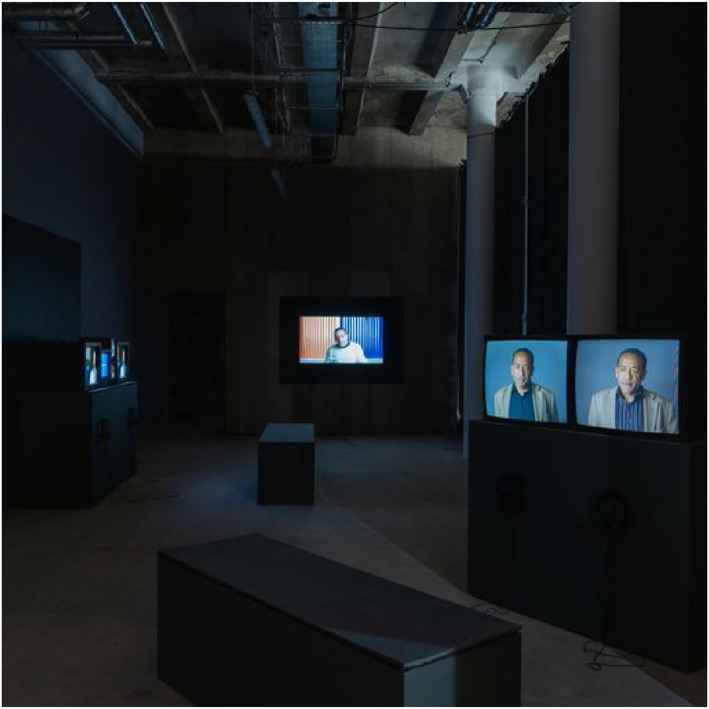
STEPHEN, installation view, Liverpool Biennial, 2023. *Credit*: © Melanie Manchot and STEPHEN Film Ltd, commissioned by Liverpool Biennial.

A multi‐channel installation presents images and sounds through the simultaneous display of multiple screens and hence troubles and subverts any linearity between time and space (see Figure 7). In single channel, and by extension cinematic screenings, the film presents visual and narrative events in a strict order that stays the same over time. This linearity forms the film's narrative logic, no matter whether documentary or fictional. This is also one of a host of conditions that allows and often invites the well‐rehearsed ‘suspension of disbelief’ which in turn is part of the immersive cinematic experience. Any multi‐channel installation, by contrast, is an active ambulatory experience that places responsibility on the viewer. This makes the story multiple and open and allows for permutations and omissions in the process. In the multi‐channel installation version of STEPHEN, the different fabulative elements consciously disrupt the capacity for the narrative to become fixed and in doing so, challenge static categories and descriptions of addiction.

The artwork version of STEPHEN combines the multi‐channel with the single‐channel across two rooms: one sculptural monitor installation ‐ the other cinema. This sets up a fabulative way of engaging with the artwork. The dissemination of STEPHEN in an art space signals that a story is not fixed nor singular but instead remains open to its differential retelling, reliving and experiencing. The creative practice of filmmaking offers multiple ways to re‐imagine relations and connections with others and an audience. As an artistic method, fabulation is a useful tool to account for a collective approach to telling stories that remain unfixed. Stepping in front of the camera to enact versions of self through fabulative imaginations becomes a process of projecting new possible modes of operating in the world that resist stigmatisation.

### 4.1 Telling stories otherwise: The fabulative becomings in STEPHEN

We are just a gang of adults pretending to be other people

Whilst multiple initiatives^2^ for the destigmatisation of drug and alcohol use focus on reframing addiction as an illness to challenge discourses of blame, the medicalisation of drug use has been widely criticised by critical drug scholars and drug user activists (Dennis et al., 2023) for depoliticising drug use and failing to take into consideration the experiences of people who use drugs. In this final section we reflect on how the methodological practice of fabulation can resist the medicalisation of addiction, engage the lived experience of people who use drugs and alcohol, and contribute to social processes of destigmatisation. Drawing on interview and observational data produced during the making of STEPHEN, in what follows we investigate the potential of fabulative methods to challenge fixed ontologies of ‘addicted’ and ‘recovered’ bodies.

The empirical data from this research emerged through observations and discussions during the pre‐production workshops and the production of STEPHEN, and a series of interviews following the completion of filming. The core group of participants consisted of 10 people who were involved in all filming and research practices, while the wider group consisted of 25 people involved with the project during its various stages. Over a 4‐year period, we followed the material *and* social lives of fabulative filmmaking practices as they produced alternative methods of telling stories. From the workshops to the filming of STEPHEN, our sociological lens focused on the method of filmmaking and the camera as a device to challenge the categorisation of our participants as ‘addicts’ and ‘recovered’. Our investigation of the methodological activity of fabulation emerged through the observation of cinematic devices and filmmaking processes as artistic practices that render visible the ambivalent relationship between fiction and reality, and in so doing produce a different doing of recovery.

### 4.2 Fabulative becomings and the ‘what if’ question

In Deleuze's (1989) description of filming fabulation, he is interested in how the fictional image of the actor overtakes the ‘real’ character, until the latter becomes invisible. In STEPHEN, the fabulative process differs from that described by Deleuze (1989), as the indiscernibility of the real and the imaginary persists throughout. The boundaries between the fictional and real image are blurred, until the two become indiscernible and enable us to think beyond the binary of a ‘true’ and ‘false’, positive and negative identity, and observe instead the emergence of new becomings. For example, the filming of ‘The Costume Fittings’ and its dissemination as an artwork in the installation at Liverpool Biennial communicated to the audience the co‐existence of the actual image of the actor with the fictional character performed. Merging scripted with non‐scripted narratives, the presentation of the actor's actual image is not a description of the individual, but an account of multiple formative encounters as described by one of the participants:

I wasn’t exactly playing myself…I included my story and a friend’s story and I ended up acting as one of my friends talking about me, about my life and their life as well so I just mixed it all up…And some people can’t differentiate and they will go oh that’s this smackhead, that drug addict talking about himself [laughing] but I’m fine with it because the more knowledge out there the better…I didn’t want to just tell my story. It’s too easy. I used a lot of reality but also a lot of imaginary. It’s me and other people’s stories.

While engaging with this filmed narrative, the audience is not invited to discern the real from the fictional, but to experience the becoming of a person, co‐shaped by personal and collective experiences, memories and scenarios. The inclusion of multiple perspectives offers the audience the opportunity to question stigmatising frameworks and explore alternative ways of understanding drug using experiences. In this process, the participants are not left exposed and vulnerable to social processes of stigmatisation, as the fabulative practices through which their narratives are produced eliminates the potential harm caused by the possibility that some people ‘*will go oh that's this smackhead…talking about himself*’ and replaces it instead with a desire to produce further knowledge on the experience of drug use. In this creative composition of multiple stories of addiction and recovery, ‘[s]tory‐telling is not an impersonal myth, but neither is it a personal fiction: it is a word in act, a speech‐act through which the character continually crosses the boundary which would separate his private business from politics, and which *itself produces collective utterances*’ (Deleuze, 1989, p. 228, emphasis in original).

An integral part of the fabulative process in the making of STEPHEN is the relationship between the recovery participants and their performance of the scripted characters. During the filming of the material presented in the art installation, as well as in the feature film, the participants had multiple opportunities to reflect on and off camera on the relationship between their actual and fictional characters. Discussing this process with them opens up new ways of challenging the stigma related spoilt identity of addiction. For some, their choice of a scripted character led to a discussion on potential alternative becomings had their life taken a different turn:

It makes more sense for me to be the [scripted character] because if I hadn’t gone off the rails…It’s quite the opposite of what I’m actually like but the same in a parallel universe [laughing]. The guy I was playing obviously was successful on some level. That’s what I think would have happened if I had gone through the usual channels…That would have led to some recovery process anyway [laughing]. If that version of me had existed, I would have ended up being fed up for having to work with idiots. I’m a failure in some respect but it’s a success to be in a position to act as this alternative self.

In this account, the participant reflects on the ‘what if’ question that appears to have informed his choice of character in the film. He sees the fictional character as a *‘successful’* version of himself, had he not ‘*gone off the rails’*. As his reflection on the fictional and actual character progresses, the binaries of success and failure gradually collapse. The long‐term ‘*success*’ of the fictional character is challenged and the actual one reframed: ‘…*it's a success to be in a position to act as this alternative self*’. Collapsing moral binaries like failure and success is of significance for the destigmatisation of the recovery process. The ‘what if’ question follows people in addiction and recovery as they attempt to navigate their life choices. In this case, rather than fantasising an alternative *‘successful’* other, that other is demythologised by being performed. Through enactment, the fictional character leaves the sphere of fantasy and becomes a reality that can be challenged and rejected, leading to a re‐evaluation of the actual character.

### 4.3 From the fictional to the real

In what follows, we share our participants' reflections on the connections that emerged when the becomings produced through filming were presented to an audience. By collectivising the experience of addiction and recovery, fabulative becomings minimised the stigma related harm potentially caused by the participants' exposure to wider, unknown audiences and enabled their re‐connection with familiar audiences who are or have been present in their lives. For some of the participants, watching the film and installation with loved ones was a way of sharing an already collective experience:

My mum, she enjoyed Stephen’s journey because she understood certain elements. She’s seen my journey. Stephen is like a vessel so to speak. You follow Ste’s journey as long you can follow your own experiences through Stephen.

For others, there was the added satisfaction of having some ‘insider’ knowledge:

I brought a few people to see it at the Tobacco Warehouse and it was nice just watching their reactions and I had a little inside knowledge of doing it… You didn’t know what was scripted and what was spontaneous, and it was really nice to see and be part of the project.

Most importantly, STEPHEN gave participants the opportunity to communicate to people that matter, but who they had disconnected from, ‘who they are today’:

It makes it easier to talk to family about what I’ve been through. I disappeared for 17 years, they didn’t know where I was and what I was doing. It’s hard to explain so it was a good way to get some connection back.

In this account, fabulation becomes the tool that makes the communication of the actual character possible, as it covers the awkward gap between the unexplained past disappearance and the current desire to re‐connect. Unlike linear narratives accounting for the stories of individuals, fabulative stories are not measured against a pre‐defined expectation for validity, where the distinction between the ‘real’ and the ‘fictional’ matters. The fabulous truths created by the participants are real *and* fictional at the same time. They are an amalgamation of past experiences, memories, support offered or withheld, and becoming with a group mediated by the camera, producing new ways of talking and connecting.

For our participants, the social process of destigmatisation emerging through the shifting narrative of drug and alcohol use and recovery, from an individual experience to a collective utterance, matters as it allows for the restoration of broken connections. Significantly, and going beyond personal gains, the practice of challenging binary categorisations and foregrounding the complexity of the participants' experiences through fabulation, calls into question the stigmatisation of drug use and recovery overall. The collectivisation emerging through fabulation is not a neutral process, but a politically charged practice that challenges the dominance of majoritarian narratives. Deleuze's (1995) interest in taking on Bergson's notion of fabulation is to give it a ‘political meaning’ (p.174). The politicisation of fabulation emerges as it challenges established fictions and highlights the relevance of minority narratives. The difference between minorities and majorities, for Deleuze, is not their size. What defines the majority, ‘is a model you have to conform to… A minority, on the other hand, has no model, it's a becoming, a process’ (p.173). Through the activity of fabulation in STEPHEN, our participants resist the majority model of addiction and recovery, and produce a collective, minority narrative that refuses to be modelled. In doing so, they rebuild connections not by restoring a spoilt, stigmatised identity, but by exposing the inability of majority recovery narratives to include their realities, and by extension the realities of all the minorities that fit no model (see Vitellone et al., 2022).

### 4.4 Fabulative ambivalence

Challenging majority narratives as stable truths, minoritarian practices embrace ambivalence, doubt and conflict. In Goffman's (1963) stigma theory, identity ambivalence emerges when the stigmatised individual obtains a close sight of the negative attributes of his or her ‘own kind’; when individuals can neither embrace their group, nor let it go. While ambivalence for Goffman is understood in relation to the tension between actual and fictional identities, we want to suggest ambivalence is not an individualised response to a stable social reality, but the outcome of contextual power structures and unrealistic social categorisations (Kierans & Bell, 2017). Elsewhere, we have highlighted the potential of embracing ambivalence as a methodological practice that can help destabilise and undo polarised oppositions of good and bad in addiction and recovery research and practice (Theodoropoulou et al., 2022). In this final empirical example, we discuss fabulation as a methodologically ambivalent tool that produces a risky relation to truth, which further destabilises the social categories of ‘addict’ and ‘recovered’.

As part of the making of STEPHEN, one of the participants decided to write and perform her story and experience of mental illness and addiction. As with all the encounters between the participants and the camera, the limits between the real and fictional were blurred. However, in this case, the realisation that the story would be exposed to the public, brought to the forefront a concern of the participant on the audience's interpretation of the fictional and real aspects of the story. Below is a fieldnote extract from the day of filming:

Today it’s the filming of [the participant’s] monologue. She decided to change it last night. She wanted to make it less personal…she was worried about the odd chance that [a family member] would watch the movie…She feels good about it today, and everyone is happy with it…We talk about the process of writing something until you realise the exposure; that it is going to be in an actual film.

Manchot's filmmaking choice, to encourage the production of fabulative stories rather than the telling of real‐life events, was seen by many participants as a liberating process, which gave them the opportunity to reflect on the complexity of their experiences, without fearing the potential assumptions made by the audience. For some of the participants, this was an opportunity to tell potential truths without feeling personally vulnerable and exposed. However, the indiscernibility of the fictional and the actual enabled by fabulation also became a source of doubt and stress when the audience's perception of the story as true or fictional came to matter. While the methodological value of fabulation as a destigmatising practice is documented through our participants' accounts on the connections enabled, there are nonetheless ethical implications to be considered. From an ethical perspective, there is a need to reflect on whether fabulative methods and artistic filmmaking practices protect participants from stigmatisation and other harms, or if, in the process of attempting to destigmatise addiction, the participants' fabulative narratives become widely available for interpretation, beyond the storytellers' control.

## 5 CONCLUSION

At the beginning I was like what can this [project] do for me? What opportunities are there for me? And now I’m like what can it do for people? The people who watch it?

The above quote is from a participant describing the potential personal benefits from their engagement with the project, and what the film STEPHEN (2023) and art installation can achieve through exposure to wider audiences. Reflecting on this shift, we conclude by rendering visible the implications of cinematic devices and fabulative methods for sociology and recovery.

Since its inception, STEPHEN has been a filmmaking project. From the onset, the filming crew, professional actors, and recovery participants, worked together to bring to life Manchot's vision of STEPHEN. Although the film and installation are co‐shaped by multiple encounters and experiences, the responsibility for the aesthetic outcome lies with the visual artist and director. The emphasis on aesthetic outcome is a significant difference between STEPHEN and art projects that emerge within recovery spaces, where the focus is on personal substance use experiences and recovery outcomes. In creative recovery focused activities taking place in recovery settings, the participants and their stories are the main protagonists and drivers of the project. In STEPHEN, the artistic engagement with the device of the camera was perceived by our participants as something new:

I like the opportunity because it’s a different approach to what I’m used to. It’s more professional. With other community projects…some of the applause is because we do well for “special people” [laughing], while this was more professional and in a sense…it’s more brutal but it makes you take it more seriously.

Within this professional setting, destigmatisation emerged through the participants' experimentations with fabulation. The final, on‐screen stories extend beyond narratives of individual experiences of substance use and recovery. They are the outcome of the co‐existence of fiction and reality, scripted scenes, and improvisations. The flexibility offered to develop these fabulative narratives was very much welcomed by the participants:

It’s good to trust the participants so instead of giving them the subject matter just giving them the idea to work with. It’s nice that you can work your own character a bit more rather than being constrained by certain stereotypes. It’s more real. There wasn’t a hero or a villain or any clichés like that.

The collective engagement with STEPHEN as primarily an artistic project was essential for our interdisciplinary collaboration to flourish. From a methodological perspective, the project advanced understandings of the uses and benefit of cinematic devices and fabulative filmmaking practices and gave our participants the opportunity to reflect on the different aspects of fabulative methods for recovery. Operating outside a majority recovery framework that foregrounds the narrative of a restored spoiled identity, participants were invited to fabulate stories whose emphasis was not on truthfulness, but on the complexity of the recovery experience. This detachment from a professional recovery context assisted with overcoming the fear of stigmatisation. Without dismissing the risk that comes with the activity of fabulation and engagement with artistic practices beyond professional recovery settings, we suggest fabulative methods open new possibilities for confronting and resisting social processes of stigmatisation that extend beyond addiction and recovery.

Importantly, the methodological potential of fabulation for processes of destigmatisation does not lie with a decontextualised replication of creative methods, but with a close attentiveness to the social assemblages that open up new life possibilities for a people to come. Our investigation of the social life of filmmaking methods involved the in situ observation and description of the transformations emerging through engagements with cinematic devices and fabulative experimentations. Rather than applying theoretical concepts to Manchot's filmmaking processes and artistic practices, we followed the stories told and the new becomings that these afforded, leading to an exploration of how the Deleuzian concept of fabulation can be operationalised for social scientific research. Whilst Deleuze's writings on fabulation derive from a retrospective engagement with films and filmmakers, thinking with Manchot's artistic practices we empirically observed the transformations emerging through fabulative stories as these were becoming, during the preproduction, production and installation of STEPHEN. The social life of fabulation emerged within a specific filmmaking assemblage, becoming over 4 years of forging connections which rendered the stories told possible. Following the social transformations emerging through the activity of filmmaking in ‘real time’, the potential of fabulation as a methodology for the advancement of destigmatisation was unravelled.

## Data Availability

The data that support the findings of this study are available on request from the corresponding author. The data are not publicly available due to privacy or ethical restrictions.

## ENDNOTES

^1^
‘Support cast’ refers to our research participants in recovery from drugs and alcohol, who engaged with the workshop activities leading to the art installation and took part in making of STEPHEN as amateur actors.

^2^
See for example, the ‘Recovery Project Florida.’
